# Ki-67 as a treatment decision modifier in breast cancer

**DOI:** 10.1093/oncolo/oyag183

**Published:** 2026-05-14

**Authors:** Amanda Dy, Jochen K Lennerz

**Affiliations:** Electrical, Computer, and Biomedical Engineering, Toronto Metropolitan University, Toronto, ON M5B 2K3, Canada; Pathology Innovations, Natera, Austin, TX 78753, United States

**Keywords:** CDK4/6 therapy, invasive ductal carcinoma, biomarker

## Abstract

Ki-67 is widely used in breast cancer; however, the fraction of patients in whom it meaningfully alters treatment decisions is not well established. Using a guideline-based approach anchored in *National Comprehensive Cancer Network (NCCN)* recommendations (v2.2026), we identified clinical scenarios in which Ki-67 directly modifies adjuvant therapy decisions and estimated their prevalence using population-level data and complementary datasets, recognizing that fully annotated datasets capturing all relevant clinicopathological variables remain limited. A back-of-the-envelope calculation suggests that only a small fraction of patients meet the combined criteria of HR+/HER2− subtype, T2 stage, grade 2 histology, and Ki-67 ≥ 20%. This estimate, compared to population-scale (*n* = 117 990 patients) and curated datasets (*n* = 1356 patients), indicates that the clinically relevant subset remains in the low single digits (3-5%). Ki-67 impacts care only in specific decision settings, particularly near clinically relevant thresholds where small analytic variation may translate into treatment changes; outside these contexts, its routine use has limited evidence for clinical utility. Its application should therefore be selective, context-dependent, and focused on scenarios in which threshold-adjacent precision is clinically consequential.

During multidisciplinary review of breast cancer specimens, it became apparent that while Ki-67 is biologically informative, the clinical contexts and its role in guiding clinical decisions are not readily available. Ki-67 is one of the most frequently ordered immunohistochemical markers, yet its impact on clinical management is often assumed rather than explicitly defined. To address this, we took a guideline-based approach using current National Comprehensive Cancer Network (NCCN) breast cancer recommendations (v2.2026). We focused on specific clinical decision points where Ki-67 is explicitly referenced as a potential determinant of treatment eligibility. This led to a pragmatic question: *when does a high Ki-67 score meaningfully alter guideline-based treatment decisions in breast cancer?*

Under current NCCN guidance, Ki-67 has a clearly defined role in HR+/HER2– early breast cancer when assessing eligibility for adjuvant CDK4/6 inhibition, specifically ribociclib, as supported by prospective, randomized evidence from the NATALEE trial that showed improved disease-free survival.^1^ Specifically, in HR+/HER2– node-negative patients with T2 (2-5 cm) tumors, and grade 2, adjuvant ribociclib may be considered when Ki-67 is ≥20% (or if genomic risk is high). In other words, a Ki-67 score of 20% becomes a decision threshold.

To address our question, we applied population-level epidemiologic data to the NCCN-guideline pathway. Approximately (70-75%) of all breast cancers are HR+/HER2–.^2^^,^^3^ Among invasive cases, ∼24.5% present as T2 tumors,^2^ and ∼42.6% as grade 2 histologies.^2^ Using a back-of-the-envelope approximation, that is, multiplying the proportions of HR+/HER2– disease (∼70-75%), T2 tumors (∼24.5%), and grade 2 histology (∼42.6%) we derive a subgroup of ∼8% of breast cancer cases as the clinically-relevant Ki-67 testing population, Within this narrowed subgroup, the distribution of Ki-67 indices has a median of ∼20-25%. This implies that roughly half of the cases lie above the 20% decision threshold (∼50% are Ki-67 high)^4^. Continuing our back-of-the-envelope calculation, multiplying the testing population (∼8%) with the Ki-67 high prevalence (∼50%) becomes ∼4%. We thereby estimated that in 4% of cases a high Ki-67 score meaningfully alters guideline-based treatment decisions in breast cancer (see Figure 1A). Importantly, this estimate assumes independence across variables and should therefore be interpreted as a heuristic approximation (Table 1). Given known biological and statistical correlations between Ki-67, tumor grade, and tumor size, the true prevalence depends on the underlying conditional distributions. Accordingly, we complement the above heuristic approximation with empirical population-level data from the Surveillance, Epidemiology and End Results Program (SEER; Table 1). These data provide a population-based anchor for the sequential selection of clinicopathological features towards a clinically relevant testing subgroup representing 6.1% of the overall population. To further assess the joint distribution of clinicopathological variables, we also used the *Molecular Taxonomy of Breast Cancer International Consortium* (METABRIC) dataset (Table 1).^5^ This cohort yielded an empirical estimate of 10.3% for the clinically relevant testing subgroup. While these datasets lack standardized Ki-67 measurements, they capture the dependence structure between tumor size, grade, and receptor status, thereby providing a complementary perspective on the co-dependence of these variables. Ki-67 is positively correlated with tumor grade and size, with higher-grade and larger tumors more likely to exhibit elevated proliferation. To account for this, we applied a plausible, conservative estimate that approximately 50% of HR+/HER2–, T2(N0M0), grade 2 tumors would meet the Ki-67 ≥ 20% threshold. This range is consistent with prospective trial data, including the monarchE study, in which ∼50% of patients in clinically enriched HR+/HER2− populations exhibited Ki-67 ≥ 20% (under centralized assessment).^6^^,^^7^ We noted that publicly available datasets either provide clinicopathological annotation without standardized Ki-67 measurements or Ki-67 measurements without full clinical context, limiting direct estimation of joint distributions. Across heuristic approximation, population-level constraints, and conditional estimates, the resulting ensemble prevalence remains ∼3-5%, indicating that the clinically relevant subset remains in the low single digits.

**Figure 1.**
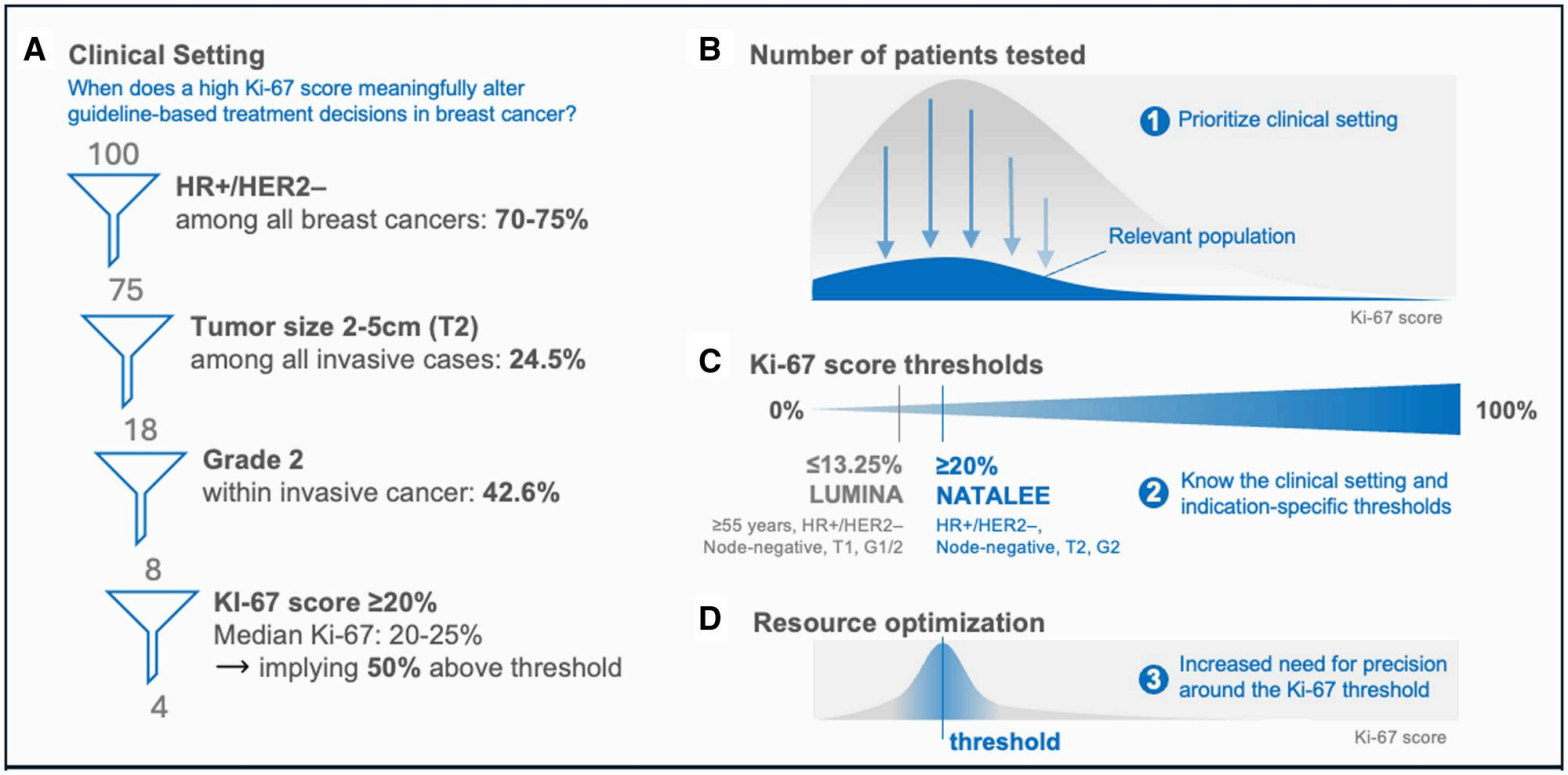
Ki-67 testing prioritization, resource optimization and example thresholds. A. Clinical setting, lead question, and prevalence estimates using published evidence (details see text and Table 1). B. Plot shows the frequency distribution of proliferation indices for two settings: all breast cancer patients ( larger reference population) and a population with selected testing in the appropriate clinical setting (=correct indication labeled ‘relevant population). Restricting testing to the clinically indicated population reduces the total number of patients tested. C. Gradient shows Ki-67 proliferation index with two exemplary Ki-67 thresholds from the LUMINA and NATALEE trials. Importantly, in LUMINA a Ki-67 score of ≤13.25% was used to omit radiotherapy whereas in NATALEE a Ki-67 score of ≥20% was used to add a CDK4/6 inhibitor. Specific thresholds and the directionality of the same biomarker (“omit vs give”) differ by indication. D. Decision-making around the threshold is most relevant and with decreased numbers of patients tested, resources can be allocated to patients with Ki-67 scores at or around the threshold.

**Table 1.** Estimation of the clinically relevant Ki-67 decision population across complementary data sources.

Approach	Back-of-the-envelope heuristic approximation^a^	Population-level query	Cohort-based analysis	Ensemble range
Source	Published Literature	SEER database	METABRIC study	
	*n* (%)	*n* (%)	*n* (%)	%
**Initial population (all cases, raw data pull)**	100	729 619 (100)	2509 (100)	
**Complete data available** ^b^	**100**	**117 990 (16.2)**	**1356 (54.0)**	
**Sequential filtering by clinicopathological features**				
** Complete data available** ^b^	**100**	**117 990 (100)**	**1356 (100)**	**100**
Hormone Receptor (HR) status: HR^+^/HER2^−^	75 (70-75)	100 239 (84.9)	988 (72.9)	70-85
Tumor stage: T2 (2–5 cm), N0 (node negative), M0 (no metastasis)	18 (24.5)	14 616 (14.6)	293 (29.7)	15-30
Histological grade 2 (G2)	8 (42.6)	7184 (49.2)	140 (47.8)	43-50
**Decision-relevant population refinement**				
** Complete data available** ^b^	**100**	**117 990 (100)**	**1356 (100)**	**100**
Clinically relevant testing population (Ki-67 indication)^c^	8 (8)	7184 (6.1)	140 (10.3)	6-10
Ki-67 ≥ 20% (threshold positive)^d^	4 (4)	3592 (3.1)	70 (5.2)	3-5
**Assessment of the Approach**				
**Advantage(s)**	Transparent assumptions; simplicity	Population anchor; real-world data	Captures dependence between variables	
**Limitation(s)**	Assumes independence; ignores co-occurrence	Data incomplete; missing Ki-67	No standardized Ki-67; selection bias	

Sequential estimation of the clinically relevant Ki-67 decision population using three complementary approaches: (i) a literature-based back-of-the-envelope heuristic approximation, (ii) a population-level query of the Surveillance, Epidemiology, and End Results (SEER) database, and (iii) a cohort-based analysis using the Molecular Taxonomy of Breast Cancer International Consortium (METABRIC) dataset.

Population-level estimates (ii) are constrained by missing data and lack of Ki-67 annotation, while cohort-based estimates (iii) capture joint variable dependence but are subject to selection bias and limited generalizability. The ensemble range reflects the convergence of these approaches, indicating that the clinically relevant subset remains in the low single digits.

a Denotes that the heuristic approach assumes independence between published clinicopathological variables.

b Denotes restriction of data to those cases with complete patient-level clinicopathological annotation.

c Denotes the clinically relevant breast cancer population that benefits from Ki-67 testing.

d The prevalence of Ki-67 ≥ 20% was estimated at 50% (see text for details). The last row shows counts and percentages relative to the total population (e.g., 3.1% of 117,990). In contrast, the percentages in the sequential filtering section of the table reflect the prevalence within each of the prior sequential filtering steps.

Bold values provide starting point (2nd row) and totals for available data before filtering (row 3) and relevant population (row 7).

Our findings in Figure 1A show that the estimate of cases where Ki-67 is explicitly invoked by guidelines is much smaller than anticipated. Assuming 100 consecutive cases would all be tested for Ki-67, for every 1 Ki-67 high case that informs guideline-based treatment, there would be 24 that do not (corresponding to 4 out of 100). Thus, the actionable role of Ki-67 is conditional rather than universal, a finding that contextualizes why Ki-67 remains as one of the most debated biomarkers in breast oncology.^8^

## Practical considerations

First, knowing the clinical setting is the basis to understand meaningful use of Ki-67 (Figure 1A). From a biological perspective, Ki-67 is an intuitively appealing marker of tumor proliferation and perceived aggressiveness, which in clinical practice often leads to its use as an additional decision-support tool beyond strictly guideline-defined contexts. The low prevalence of guideline-defined evidence-based indications where Ki-67 meaningfully alters management argues against routine Ki-67 testing in all breast cancer cases. This practice supports diagnostic stewardship, tissue preservation, and cost containment. For example, in some laboratories, Ki-67 is proposed as part of multi-marker panels.^9^^,^^10^ While technically possible, our analysis illustrates the implied extra effort of >90% without guideline-compliant actionability (Figure 1B).

Second, for these specific clinical settings and indications, it is of crucial importance to know the decision threshold (Figure 1C). When Ki-67 is used to modify treatment decisions, a one percentage point shift in the score can have substantial consequences. For example, a Ki-67 score of ≤13.25% has been evaluated as a criterion to de-escalate local therapy in the LUMINA cohort study.^11^ In this context, a difference of less than one percentage point can meaningfully inform clinical decisions. By contrast, the same degree of variability may be clinically inconsequential when Ki-67 is reported as 14% and the relevant decision threshold is 20%. In these settings, analytic and interpretive variance become clinical variance. Therefore, knowing the clinical setting and decision threshold is important to avoid unintentional imprecision. Accordingly, the funnel presented here reflects scenarios in which Ki-67 itself functions as a decision-modifying variable, rather than the overall prevalence of adjuvant CDK4/6 inhibitor use.

Third, knowing the relevant decision threshold has another important practical implication: it allows precision efforts to be focused where they matter most, namely, in cases with Ki-67 scores near the decision threshold (Figure 1C). Precision in this context refers to prioritizing time, expertise, and quality controls for cases in which small differences in Ki-67 scoring could alter management. While standardized, visual, and careful assessment is one option, visual scoring alone may lack sufficient reproducibility around clinical thresholds. Accordingly, resource optimization may also include digital image analysis and AI-enabled quantification tools. These can aid in objective Ki-67 assessment and help mitigate reproducibility concerns,^12^ especially in cases with Ki-67 scores near the relevant decision threshold.

Fourth, complete and well-annotated clinicopathological data are a prerequisite for the clinically meaningful use of Ki-67. In our analysis, even seemingly straightforward classifications (ie, tumor stage, nodal status, grade, and receptor status) were not consistently available, leaving insufficient context for clinical use of Ki-67. More liberal Ki-67 ordering practices cannot substitute for complete staging, grading, and receptor assessment. This aligns with ongoing efforts by professional organizations such as the *College of American Pathologists*, the *American Society for Clinical Pathology*, and the *International Ki-67 in Breast Cancer Working Group*, which emphasize standardized assessment and context-specific use of Ki-67. Greater attention to data integrity will be essential to translate biomarker insights into reliable clinical practice.

Importantly, this analysis does not argue against Ki-67 as a biomarker, but for its meaningful and context-specific use. Ki-67 remains clinically relevant for luminal subtype refinement, prognostic enrichment, trial eligibility, and assessment of treatment response in the neoadjuvant setting, where changes in Ki-67 correlate with outcome.^13^ However, its use as a decision tool is limited by analytic and interpretive variability, including pre-analytic factors, scoring methods, inter-observer reproducibility, and inconsistent decision thresholds; limitations repeatedly highlighted by expert reviews and professional societies^13^^,^^14^ contributing to ongoing controversy and, in some settings, removal from regulatory labels. Differences in how Ki-67 is applied across adjuvant CDK4/6 inhibitor programs further emphasize the need for standardized assessment and interpretation.^15^

Ki-67 plays distinct roles across trials, regulatory authorization, and guideline implementation. Historically, Ki-67 has been widely used as a prognostic biomarker reflecting tumor proliferation. With the emergence of prospective trials evaluating adjuvant CDK4/6 inhibitors, including the monarchE^7^ and NATALEE^1^ trials, Ki-67 ≥ 20% was incorporated to define high-risk subgroups. In 2021, the U.S. Food and Drug Administration (FDA) approved adjuvant abemaciclib for HR+/HER2− early breast cancer with high-risk features, requiring Ki-67 ≥ 20% as a companion diagnostic for treatment selection.^16^ This designation facilitated initial regulatory approval and clinical adoption by providing a measurable threshold for patient selection (=market entry). However, in 2023,^17^ the FDA removed the Ki-67 companion diagnostic requirement following longer-term analyses of monarchE demonstrating benefit independent of Ki-67 status,^18^ alongside ongoing concerns regarding assay variability and reproducibility.^19–21^ As a result, Ki-67 has transitioned to a context-specific decision modifier reflecting that biomarker roles evolve with clinical, evidentiary, and regulatory context.^22^ Thereby, Ki-67 in breast cancer exemplifies how a (prognostic) biomarker can be elevated to a companion diagnostic for treatment selection and market entry, then later repositioned as evidence and practice evolve. Similarly, regulatory approvals evolve with emerging scientific evidence in accordance with principles of regulatory science.^17^

Notably, in assembling these estimates, we were struck by the limited availability of datasets with complete patient-level annotation. In population-scale datasets, substantial missingness or the need for sequential restriction markedly reduces the analyzable cohort.^23^ As a result, we were unable to identify a single, readily available dataset that captures these variables jointly at the patient level to directly assess their dependence structure. This limitation has broader implications, including for AI/ML applications, where robust methods to address sparse and/or systematically missing data remain an unmet need.

In an era of increasingly resource-constrained oncology care, even familiar biomarkers benefit from periodic re-examination, not to diminish their value, but to align their use with demonstrable, guideline-anchored clinical impact.
